# A combination of molecular and clinical parameters provides a new strategy for high-grade serous ovarian cancer patient management

**DOI:** 10.1186/s12967-022-03816-7

**Published:** 2022-12-21

**Authors:** Melissa Bradbury, Eva Borràs, Marta Vilar, Josep Castellví, José Luis Sánchez-Iglesias, Assumpció Pérez-Benavente, Antonio Gil-Moreno, Anna Santamaria, Eduard Sabidó

**Affiliations:** 1 Centre de Regulació Genòmica, Barcelona Institute of Science and Technology (BIST), Dr Aiguader 88, 08003 Barcelona, Spain; 2 Universitat Pompeu Fabra, Dr Aiguader 88, 08003 Barcelona, Spain; 3 Biomedical Research Group in Gynecology, Vall d’Hebron Institut de Recerca, Universitat Autònoma de Barcelona, Vall, d’Hebron Barcelona Hospital Campus, Passeig Vall d’Hebron 119-129, 08035 Barcelona, Spain; 4 Department of Gynecology, Hospital Universitari Vall d’Hebron, Vall d’Hebron Barcelona Hospital Campus, Passeig Vall d’Hebron 119-129, 08035 Barcelona, Spain; 5 Department of Pathology, Hospital Universitari Vall d’Hebron, Vall d’Hebron Barcelona Hospital Campus, Passeig Vall d’Hebron 119-129, 08035 Barcelona, Spain; 6 Centro de Investigación Biomédica en Red (CIBERONC), Instituto de Salud Carlos III, Avenida de Monforte de Lemos 3-5, 28029 Madrid, Spain; 7 Cell Cycle and Cancer Laboratory, Biomedical Research Group in Urology, Vall Hebron Institut de Recerca, Vall d’Hebron Barcelona Hospital Campus, Universitat Autònoma de Barcelona, Passeig Vall d’Hebron 119-129, 08035 Barcelona, Spain

**Keywords:** High-grade serous ovarian cancer, Proteomics, Biomarker, Prediction, Treatment

## Abstract

**Background:**

High-grade serous carcinoma (HGSC) is the most common and deadly subtype of ovarian cancer. Although most patients will initially respond to first-line treatment with a combination of surgery and platinum-based chemotherapy, up to a quarter will be resistant to treatment. We aimed to identify a new strategy to improve HGSC patient management at the time of cancer diagnosis (HGSC-1LTR).

**Methods:**

A total of 109 ready-available formalin-fixed paraffin-embedded HGSC tissues obtained at the time of HGSC diagnosis were selected for proteomic analysis. Clinical data, treatment approach and outcomes were collected for all patients. An initial discovery cohort (n = 21) were divided into chemoresistant and chemosensitive groups and evaluated using discovery mass-spectrometry (MS)-based proteomics. Proteins showing differential abundance between groups were verified in a verification cohort (n = 88) using targeted MS-based proteomics. A logistic regression model was used to select those proteins able to correctly classify patients into chemoresistant and chemosensitive. The classification performance of the protein and clinical data combinations were assessed through the generation of receiver operating characteristic (ROC) curves.

**Results:**

Using the HGSC-1LTR strategy we have identified a molecular signature (TKT, LAMC1 and FUCO) that combined with ready available clinical data (patients’ age, menopausal status, serum CA125 levels, and treatment approach) is able to predict patient response to first-line treatment with an AUC: 0.82 (95% CI 0.72–0.92).

**Conclusions:**

We have established a new strategy that combines molecular and clinical parameters to predict the response to first-line treatment in HGSC patients (HGSC-1LTR). This strategy can allow the identification of chemoresistance at the time of diagnosis providing the optimization of therapeutic decision making and the evaluation of alternative treatment strategies. Thus, advancing towards the improvement of patient outcome and the individualization of HGSC patients’ care.

**Supplementary Information:**

The online version contains supplementary material available at 10.1186/s12967-022-03816-7.

## Background

High-grade serous carcinoma (HGSC) remains the most common and deadly subtype of ovarian cancer, due to its diagnosis at advanced stages in over 80% of cases [1]. The standard of care of advanced stage HGSC is a combination of cytoreductive surgery and platinum-based chemotherapy (i.e. carboplatin and paclitaxel). Cytoreductive surgery can be performed at the time of cancer diagnosis or can be delayed after 3 or 4 cycles of neoadjuvant chemotherapy [2]. Recently, the use of a targeted management approach (i.e. anti-angiogenic agents and PARP inhibitors) has been introduced for specific subgroups of patients such as those with BRCA mutations [3, 4]. Although the results on BRCA mutation status can be available within 2–3 weeks from diagnosis in some referral oncological centres, in most clinical settings worldwide it is not available until first-line treatment has already commenced. Although HGSC is considered to be a platinum-sensitive disease, ~ 20–30% of patients fail to respond or experience disease recurrence within 6 months of completing chemotherapy [5]. In clinical practice, these patients are considered to be resistant to platinum, and second-line treatment is usually based on non-platinum agents (e.g. gemcitabine, pegylated liposomal doxorubicin, topotecan or weekly paclitaxel) and control of symptoms [2, 6]. Efforts to establish biological stratification profiles in HGSC are mainly being focused on genomic and transcriptomic markers [7–10], while the evaluation of protein markers to assess treatment response and/or survival in HGSC tissue samples remain scarce [11–13]. Beyond their clinical relevance as biological endpoints and drug targets, proteins are markers widely used in clinical tests for disease diagnosis and prognosis [13, 14]. In addition, the use of proteomic approaches (i.e. mass spectrometry) enable the systematic interrogation of proteomes from complex clinical samples which can later be translated into immunoassays for clinical use [15, 16]. Despite the numerous studies being carried out to identify novel protein biomarkers in ovarian cancer, there are currently no validated markers used in the clinic to predict response to first-line treatment and guide the management of patients with newly diagnosed HGSC. In this work, we present a new strategy that combines molecular and routine clinical parameters to anticipate the response to first-line treatment in HGSC patients (HGSC-1LTR). Prediction of those patients less likely to respond to first-line chemotherapy allows alternative treatment strategies to be considered at the time of cancer diagnosis (i.e. non-platinum drugs) thus improving patients’ response and survival. It also optimizes therapeutic decision making and allows an individualized management strategy by avoiding the use of futile treatments, thus improving patients’ quality of life. This strategy is therefore of special relevance for patients with advanced HGSC for whom a predictive method is lacking. Thus, in this work we aim to identify a protein biomarker signature able to predict response to first-line treatment in patients with newly diagnosed HGSC.

## Methods

### Patient cohorts

A total of 109 patients with newly diagnosed advanced stage HGSC managed at the Gynecological Oncology Unit at the Hospital Vall d’Hebron (Barcelona, Spain) between 1996 and 2017 were included in the study. Out of these, a fifth (21 patients) were randomly selected for the discovery cohort and four fifths (88 patients) for the verification cohort. Patients were classified according to their treatment-free interval to platinum (TFIp) into two main groups: TFIp < 6 months (chemoresistant) and TFIp > 6 months (chemosensitive). For the discovery cohort, the chemosensitive group was subdivided into those patients who developed recurrence (chemosensitive with TFIp > 6 months) and those who did not recur (chemosensitive with no recurrence) in order to categorise the subgroup of patients with better response to chemotherapy. The characteristics of all patients included in the discovery and verification phases are summarized in Tables 1 and 2 and Additional file 1: Table S1 and Additional file 2: Table S2.

**Table 1:** Summary of the clinical characteristics of the patient groups included in the discovery cohort

	Chemoresistant with TFIp < 6 m (n = 7)	Chemosensitive with TFIp > 6 m (n = 7)	Chemosensitive with no recurrence (n = 7)
*Age (years)*			
Average (range)	67 (53–77)	58 (49–69)	60 (43–70)
*Menopausal status (n, %)*			
Premenopausal	0	2 (29%)	1 (14%)
Postmenopausal	7 (100%)	5 (71%)	6 (86%)
*Stage (n, %)*			
IIIC	6 (86%)	6 (86%)	7 (100%)
IV	1 (14%)	1 (14%)	0
*Serum CA125 levels at diagnosis (U/mL)*			
Average (range)	1944 (809–5500)	2444 (126–6992)	1051 (100–2856)
*TFIp (months)*			
Average (range)	3 (1–5)	28 (11–67)	NA
*Primary treatment (n, %)*			
Cytoreductive surgery	4 (57%)	5 (71%)	4 (57%)
Neoadjuvant chemotherapy	3 (43%)	2 (29%)	3 (43%)
*Status (n, %)*			
Alive	0	1 (14%)	7 (100%)
Dead	7 (100%)	6 (86%)	0

TFIp: treatment-free interval to platinum

**Table 2:** Summary of the clinical characteristics of the patient groups included in the verification cohort

	Chemoresistant (n = 25)	Chemosensitive (n = 63)
*Age (years)*		
Average (range)	63 (49–81)	59 (37–84)
*Menopausal status (n, %)*		
Premenopausal	1 (4%)	18 (29%)
Postmenopausal	24 (96%)	45 (71%)
*Stage (n, %)*		
IIIA	0	2 (3%)
IIIB	1 (4%)	6 (10%)
IIIC	16 (64%)	45 (71%)
IVA	3 (12%)	4 (6%)
IVB	5 (20%)	6 (10%)
*Serum CA125 levels at diagnosis (U/mL)*		
Average (range)	2246 (161–10,548)	1385 (20–19,007)
*TFIp (months)*		
Average (range)	3 (1–5)	13 (6–63)
*Primary treatment (n, %)*		
Cytoreductive surgery	7 (28%)	44 (70%)
Neoadjuvant chemotherapy	18 (72%)	19 (30%)
*Status (n, %)*		
Alive	0	31 (49%)
Dead	25 (100%)	32 (51%)

TFIp: treatment-free interval to platinum

### Formalin-fixed paraffin-embedded tissue samples

Formalin-fixed paraffin-embedded (FFPE) tissue samples were obtained from the Pathology Department human tissue repository at the Hospital Vall d’Hebron (Barcelona, Spain). All cases corresponded to HGSC tumour biopsies obtained at the time of diagnosis. Samples were registered, processed and fixed following the hospital standard operating procedures. Pathological and clinical data from all gynaecological registered samples were manually reviewed to ensure that all patients included in the study met the following criteria: (a) newly diagnosed advanced stage HGSC (stages III and IV), (b) treatment with primary surgery and six cycles of carboplatin and paclitaxel chemotherapy, (c) follow-up until disease recurrence or at least five years after treatment completion. The histopathological features of the selected samples were then reviewed by an experienced pathologist to confirm the diagnosis and tumour content. Areas containing 80% or more tumour with no areas of necrosis were selected for serial sectioning and further sample processing.

### Discovery cohort

#### Sample preparation for proteomic analysis

An initial cohort of 21 patients were randomly selected for biomarker discovery from whom FFPE tumour samples were collected. For FFPE sample preparation we adapted a recently described workflow capable of yielding substantial amounts of peptides for quantification by proteomic analysis with high reproducibility [17]. FFPE tissues were cut in five serial sections of 10 µm thick using a microtome. A clean blade was used for sectioning each tissue sample separately. Tissue sections were deparaffinated in 1 ml xylene (3 min at 50 °C) and washed twice with 1 ml absolute ethanol. Ethanol was removed completely and sections were left to air-dry.

#### Data analysis

Acquired data were analysed using the Proteome Discoverer software suite (v.2.0, Thermo Fisher Scientific) and peptides were identified using the Mascot search engine (v.2.5.1, Matrix Science). Data were searched against the Swiss-Prot human protein database (as in October 2017, 20,239 entries) plus a list of common contaminants (148 entries) [18]. The precursor ion mass tolerance was 7 ppm at the MS1 level, and up to three missed cleavages for trypsin were allowed. The fragment ion mass tolerance was set to 0.5 Da and methionine oxidation was set as variable modification. The identified peptides were filtered by 5% FDR. Peptide areas were obtained using the “Precursor Ions Area Detector” module in the Proteome Discoverer software suite (v.2.0, Thermo Fisher Scientific). Protein abundance in each condition was estimated using the average of the three most intense peptides per protein group (Additional file 3: Table S3). Data were log2-transformed, normalized by equalised median and quantified using the MSstats R software package v.3.8.2 [19]. Only those proteins with quantitative values in at least 4 out of 7 patients per group were considered in the group comparison analysis. Changes in protein abundance between groups were compared using two-sided *t* test analysis followed by correction for multiple testing [20]. Changes were considered significant with a q-value below 0.05.

All significant differentially expressed proteins were included in the analysis. Additional proteins were selected according to at least one of the following criteria: (a) were present in at least 4 patients in one group and completely absent in the comparison group, (b) were either completely present or completely absent in the chemoresistant group, (c) had been previously reported in the literature as potential predictive markers in ovarian cancer. Nine proteins were included using these criteria, three of which corresponded to proteins drawn from the literature (i.e. CT45, CDK1 and CLDN3) [11, 21–23].

### Verification cohort

#### Sample preparation for proteomic analysis

A cohort of 88 patients were randomly selected for biomarker verification from whom FFPE samples were collected. FFPE tissues were cut in 5 serial sections of 10 µM thick using a microtome. A clean blade was used for sectioning each tissue sample separately. Tissue sections were deparaffinated in 1 ml xylene (3 min at 50 °C) and washed twice with 1 ml absolute ethanol. Ethanol was removed completely and sections were left to air-dry. Samples were resuspended in lysis buffer (40 mM TrisHCl, 1% SDS, pH 8.2) and left to incubate at 99 °C for 30 min and 80 °C for 2 h in a thermomixer. Following centrifugation (20 min, RT, 15,000*g*) the supernatant was quantified using BCA protein assay. Protein extracts were diluted 20 times with 50 mM ammonium bicarbonate for digestion with trypsin (1:50 w:w, 37 °C, 8 h, Promega cat # V5113). Detergent was removed from protein digests using the HIPPR™ Detergent Removal Spin Column Kit (Thermo Scientific, PN 88305) following manufacturer instructions. Peptide mix was acidified with formic acid 5% and desalted with a MicroSpin C18 column (The Nest Group, Inc). Isotopically-labelled peptides (^13^C_6_,^15^N_2_-Lys and ^13^C_6_,^15^N_4_-Arg, Pepotec Peptides, Thermo Fisher Scientific) were spiked in the peptide mixtures and used as internal standard for quantification by parallel reaction monitoring (PRM). A total of 30 proteins and 59 peptides were selected for PRM from 88 samples (25 chemoresistant and 63 chemosensitive).

#### Parallel reaction monitoring

Up to two unique peptides per protein were selected for targeted protein quantification, prioritizing those peptides that had been previously observed in the discovery cohort. For each selected peptide, an isotopically-labelled peptide (^13^C_6_,^15^N_4_-Arginine, and ^13^C_6_,^15^N_2_-Lys) was spiked in the samples and used as an internal standard for quantification by Parallel reaction monitoring (PRM). The amount of internal standard peptide to be spiked in each sample was evaluated using dilution curves and the final concentration was chosen based on the following criteria: (a) to be within the concentration range in which a linear response of the peptide was observed and, (b to have an area as close to the endogenous peptide area as possible. One microgram of digested sample was analysed by PRM using an Orbitrap Fusion Lumos (Thermo Fisher Scientific) coupled to an EASY-nanoLC 1000 UPLC system (Thermo Fisher Scientific) with a 50-cm C18 chromatographic column. Peptide mixes were separated with a chromatographic gradient starting at 95% buffer A and 5% buffer B with a flow rate of 300 nL/min and going up to 25% buffer B and 75% A in 52 min and to 40% B and 60% A in 8 min (Buffer A: 0.1% formic acid in water and Buffer B: 0.1% formic acid in acetonitrile). The Orbitrap Fusion Lumos was operated in positive ionization mode with an EASY-Spray nanosource at 2.4 kV and at a source temperature of 275 °C. A scheduled PRM method was used for data acquisition with a quadrupole isolation window set to 1.4 m*/z* and MS2 scans over a mass range of *m/z* 340–950, with detection in the Orbitrap mass analyser at a 60 K resolution. MS2 fragmentation was performed using HCD fragmentation at normalised collision energy of 30%, the AGC was set at 50,000 and the maximum injection time at 118 ms. The size of the scheduled window was 6 min and the maximum cycle time was 2.8 s. All data was acquired with XCalibur software v.3.0.63. QCloud was used to control instrument longitudinal performance during the project [24].

#### Data analysis

Product ion chromatographic traces corresponding to the targeted precursor peptides were evaluated with Skyline software v.4.2 [25] based on: (a) the number of detected traces, (b) co-elution of endogenous traces, (c) co-elution of endogenous and internal standard peptides, (d) correlation of the trace relative intensities between endogenous and internal standard peptides and, (e) expected retention time. Those transitions showing interferences on the PRM traces were discarded. Measurements which were considered to be under the limit of detection were replaced with an estimation of the background value. Peptides who did not fulfil all the above criteria were removed from the study (seven peptides in total). In addition, eight samples were also discarded due to sample quality. In all, we consistently quantified a total of 52 peptides corresponding to 29 proteins across 80 samples. Peak areas were obtained for each production and data were log2-transformed prior to normalization and statistical analysis (Additional file 5: Table S4). Normalization relied on internal isotopically-labelled standard peptides which were used to equalise the median abundance of the internal standard peptides across all runs and then shift all endogenous areas in a run by a same amount. Protein abundance estimates were performed with the software package MSstats 3.14.1 [19]. Missing quantification values were imputed with a minimum estimated log2-transformed abundance for a given protein across runs.

### Predictive analysis

For predictive analysis 29 proteins and 29 peptides (best peptide for each protein) were used (Additional file 6: Table S5). The final verification cohort (n = 80) was divided into a training subset and a validation subset with a 8:10 ratio. Within the training set, the abundance of each protein was fitted in a logistic regression model between chemoresistant and chemosensitive patients and the classification ability of each protein was evaluated by the area under the curve (AUC) of a receiver operating characteristic. The protein with the highest AUC was selected as the first classifier. Most discriminative proteins were repeatedly added to the classifier one by one as long as their combination resulted in an increase in AUC value higher than 0.02. The best protein combination in the training subset was fitted in a logistic regression model and was applied to the validation subset. The procedure from division into training and validation set to fitting of the logistic model with the best classification signature was repeated 500 times to assess the reproducibility of classification ability. The final consensus model was comprised of the combination of proteins which were selected with higher frequencies in the 500 repeats [26]. The pROC package in R was used to draw ROCs, calculate AUCs and other predictive performance data including sensitivity and specificity at the optimal cut-off threshold (Youden J Index) for discrimination between groups [27].

## Results

### Identification of protein biomarker candidates from formalin-fixed paraffin embedded HGSC tissues

Our first aim was to identify potential protein biomarker candidates able to predict response to first-line treatment in patients with newly diagnosed HGSC. To this intent, we performed a discovery proteomic analysis of FFPE tumour samples from a cohort of 21 patients diagnosed with advanced stage HGSC. This analysis identified proteins that differed in abundance in relation to the patients’ response to chemotherapy with carboplatin and paclitaxel. We divided the cohort according to the time of disease recurrence into chemoresistant and chemosensitive [28] (see “Materials and Methods” section). Table 1 and Additional file 1: Table S1 summarize the clinical characteristics of the groups of patients included in the discovery cohort.

**Fig. 1:**
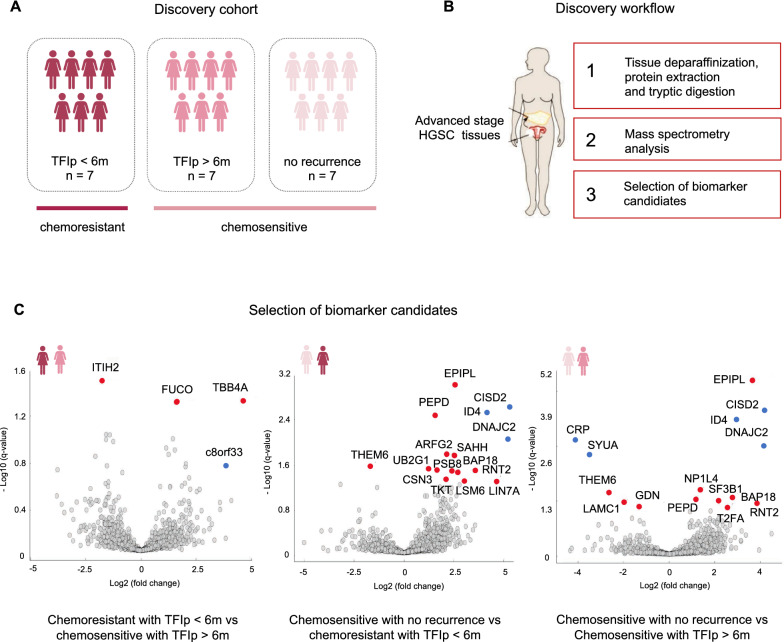
Identification of protein biomarker candidates using discovery proteomics. **A** Graphical abstract of the patient groups and number of samples included in the discovery phase. **B** Overview of the proteomics workflow used in the discovery phase. HGSC indicates high-grade serous ovarian cancer tumours. **C** Volcano plots of the pairwise comparisons between the three groups included in the discovery phase (chemoresistant with TFIp < 6 months, chemosensitive with TFIp > 6 months and chemosensitive with no recurrence). Highlighted in red are the differentially expressed proteins and highlighted in blue are the proteins present in at least four patients in one group and completely absent in the other group

We quantified a total of 6813 proteins in our discovery dataset (Fig. 1B). Only those proteins identified in at least 4 out of 7 patients in each group (2441 proteins on average) were considered for quantitative analysis and statistical group comparison evaluation (Additional file 4: Fig. S1A). We identified 27 differentially expressed proteins between the three groups (chemoresistant with TFIp < 6 months, chemosensitive with TFIp > 6 months, and chemosensitive with no recurrence) (q-value < 0.05) (Fig. 1C). Of these, 21 proteins showed changes in abundance, whilst 6 proteins were present in at least four patients in one group and completely absent in the comparison group. These six proteins include CISD2, CRP, DNAJC10, ID4, SYUA and protein C8orf33. Additionally, three proteins previously shown to be associated to ovarian cancer prognosis were added from the literature (i.e. CT45, CDK1 and CLDN3) [11, 21–23]. Hence, a total of 30 proteins were selected as potential protein biomarker candidates for verification by targeted proteomics in an independent cohort of HGSC patients.

### Verification of protein biomarker candidates from formalin-fixed paraffin- embedded HGSC tissues

Protein verification acts as a bridging phase capable of overcoming the gap between biomarker discovery and validation [15, 29]. In the verification cohort we aimed to accurately quantify the candidate biomarkers obtained by discovery proteomics as a basis for our predictive analysis circumscribing the intended use of our potential predictive biomarker signature to the classification of patients into the chemoresistant and chemosensitive groups.

**Fig. 2:**
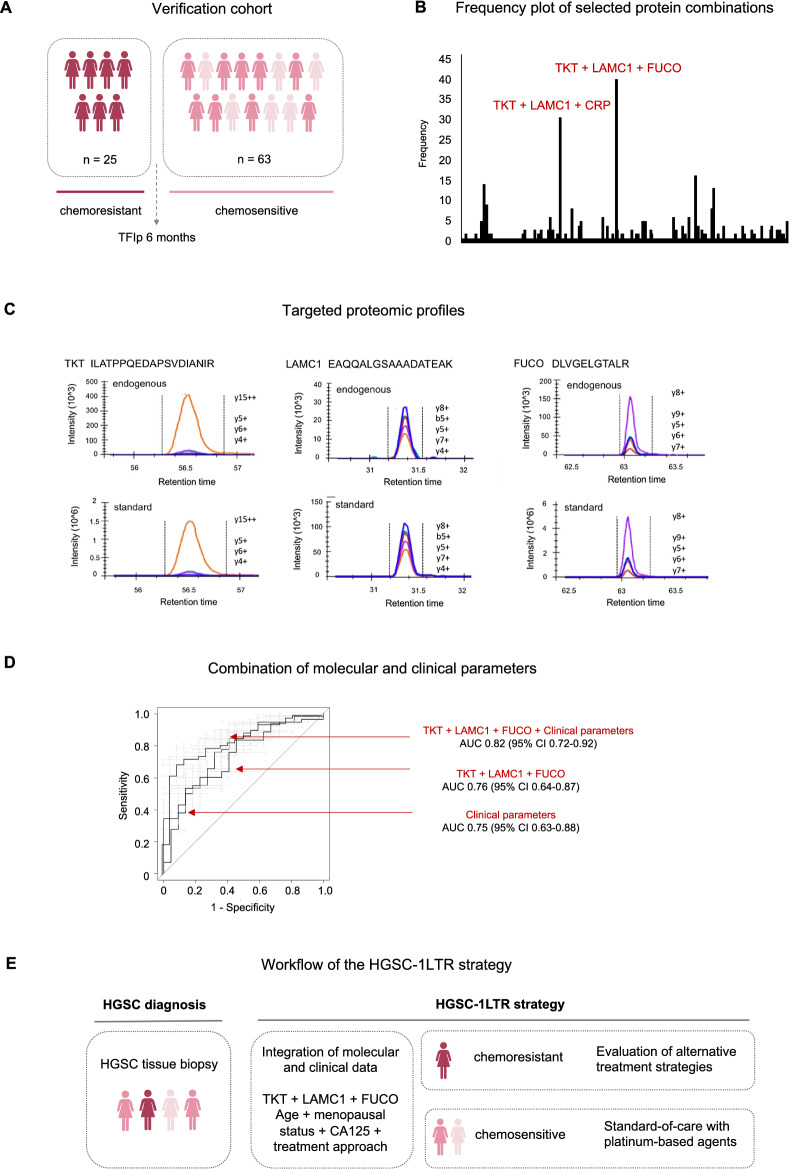
Predicted proteins able to discriminate between chemoresistant and chemosensitive patients. **A** Graphical abstract of the patient groups included in the verification cohort. **B** Frequency plot representing the number of times a protein combination was selected over 500 iterations. **C** Targeted proteomic profiles (fragment extracted ion chromatograms) corresponding to the endogenous peptides and their internal standards for proteins TKT, LAMC1 and FUCO. **D** Receiver operating curves corresponding to the predictor formed by the combination of proteins TKT + LAMC1 + FUCO and clinical parameters including age, menopausal status, CA125 levels at diagnosis and decision to treat with either primary cytoreductive surgery or neoadjuvant chemotherapy. The protein combination TKT + LAMC1 + FUCO (AUC from 0.76; 95% CI 0.64–0.87) in combination with clinical data (AUC 0.75; 95% CI 0.63–0.88) increased the AUC value to 0.82 (95% CI 0.72–0.92), *p* = 0.09. **E** Workflow of the HGSC-1LTR strategy. Identification of chemoresistance can facilitate the study of alternative treatments to improve patient outcome. Patients classified as chemosensitive could undergo the standard of care with platinum-based agents

We verified the biomarker candidates selected from the discovery cohort using targeted proteomics. In particular, we used parallel reaction monitoring (PRM) in an independent cohort of 88 FFPE tumour samples to assess the ability of the selected biomarker candidates to predict patients’ response to first-line treatment. In this verification cohort, we also sought to define the potential clinical applicability of a protein biomarker signature. Because the intended use is the prediction of response to first-line treatment in patients with newly diagnosed advanced stage HGSC, we limited our predictive analysis to two groups based on the TFIp cut-off of 6 months and therefore divided our verification cohort into chemoresistant (TFIp < 6 months) (n = 25) and chemosensitive (TFIp > 6 months) (n = 63) groups (Fig. 2A). The characteristics of the patient groups included in the verification phase are summarized in Table 2 and Additional file 2: Table S2.

FFPE tumour samples were processed in the same manner as in the discovery phase. In order to quantify the protein biomarker candidates by PRM, we selected a specific subset of representative peptides which were used as surrogates for each protein candidate. We chose 1 or 2 peptides per protein based on their uniqueness to the given target, their chromatographic and mass spectrometry performance and their stability [30]. Using these criteria we selected a total of 59 peptides corresponding to the 30 protein candidates for quantification by PRM (Table 3). Internal standard peptides were used to guide the identification of the endogenous peptides and to accurately quantify them in the tumour samples. These standards consisted of identical peptide sequences as the endogenous with the C-terminus amino acid isotopically labelled which were spiked into each sample for analysis (Additional file 4: Fig. S1B). The technical variability of the assay was calculated as a coefficient of variation (CV) showing a median CV of 3%, being the highest value 26% (Additional file 4: Fig. S1C).

**Table 3:** List of the 30 proteins and 52 peptides selected for Parallel Reaction Monitoring measurement

Accession	Protein	Peptide
Q8N6H7	ARFG2 _ADP-ribosylation factor GTPase-activating protein 2	EVDAEYEAR
		LAYQELQIDR
Q8IXM2	BAP18 _Chromatin complexes subunit BAP18	VYEDSGIPLPAESPK
		VGEIFSAAGAAFTK
Q9H7E9	C8orf33 _UPF0488 protein C8orf33	AAAYSAQVQPVDGATR
		LPGPVSSAR
P06493	CDK1 _Cyclin-dependent kinase 1	SPEVLLGSAR
		NLDENGLDLLSK
Q8N5K1	CISD2 _CDGSH iron-sulfur domain-containing protein 2	LPVPESITGFAR
		DSLINLK
O15551	CLD3 _Claudin-3	STGPGASLGTGYDR
		VVYSAPR
Q9UNS2	CSN3 _COP9 signalosome complex subunit 3	ASALEQFVNSVR
		AMDQEITVNPQFVQK
P02741	CRP _C-reactive protein	ESDTSYVSLK
		QDNEILIFWSK
P0DMU7	CT45A6 _Cancer/testis antigen family 45 member A6	VAVDPETVFK
		IFEMLEGVQGPTAVR
Q8IXB1	DJC10 _DnaJ homolog subfamily C member 10	NFQEEQINTR
		ILYDILAFAK
P58107	EPIPL _Epiplakin	AEAEAGSPRPDPR
		ALQQGLVGLELK
P04066	FUCO _Tissue alpha-L-fucidase	DLVGELGTALR
		FFHPEEWADLFQAAGAK
P07093	GDN _Glia-derived nexin	ASAATTAILIAR
		IEVSEDGTK
P47928	ID4 _DNA-binding protein inhibitor ID-4	TPLTALNTDPAGAVNK
P19823	ITIH2 _Inter-alpha-trypsin inhibitor heavy chain H2	LSNENHGIAQR
		IQPSGGTNINEALLR
P11047	LAMC1 _Laminin subunit gamma-1	NTIEETGNLAEQAR
		EAQQALGSAAADATEAK
O14910	LIN7A _Protein lin-7 homolog A	IIPGGVAER
		ATVAAFAASEGHSHPR
P62312	LSM6 _U6 snRNA-associated Sm-like protein LSm6	LNSGVDYR
		GNNVLYISTQK
Q99733	NP1L4 _Nucleome assembly protein 1-like 4	AAATAEEPDPK
		VLAALQER
P12955	PEPD_Xaa-Pro dipeptidase	IDEPGLR
		LPASHATWMGK
P28062	PSB8 _Proteasome subunit beta type-8	FQHGVIAAVDSR
		ASAGSYISALR
O00584	RNT2 _Ribonuclease T2	VYGVIPK
		ELDLNSVLLK
P23526	SAHH_Adenylhomocysteinase	GISEETTTGVHNLYK
		VADIGLAAWGR
O75533	SF3B1 _Splicing factor 3B subunit 1	THEDIEAQIR
		WDQTADQTPGATPK
P37840	SYUA _Alpha-synuclein	EGVVHGVATVAEK
		TVEGAGSIAAATGFVK
P35269	T2FA _General transcription factor IIF subunit 1	LDTGPQSLSGK
		EFRPEDQPWLLR
Q8WUY1	THEM6 _Protein THEM6	LLEPFEVR
		AFYLEAR
P29401	TKT _Transketolase	SVPTSTVFYPSDGVATEK
		ILATPPQEDAPSVDIANIR
P04350	TBB4A _Tubulin beta-4A chain	EVDEQMLSVQSK
		INVYYNEATGGNYVPR
P62253	UB2G1 _Ubiquitin-conjugating enzyme E2 G1	FITEIWHPNVDK
		TELQSALLLR

### Identification of a protein signature able to classify patients into chemoresistant and chemosensitive groups

The final step of this verification cohort was aimed at defining protein combinations able to classify patients diagnosed with advanced stage HGSC into those who will respond to first-line chemotherapy treatment with carboplatin and paclitaxel and those who will not. To this intent, we performed a logistic regression model to select and evaluate those proteins able to correctly classify patients into chemoresistant and chemosensitive groups, both individually and in combination. Details of the cross-validation analysis can be found in the corresponding methods section. In brief, the final patient cohort (n = 80) was randomly divided into a training set and validation set. The classification power of each protein was first evaluated in the training set by a logistic regression model and additional proteins were then added into the best protein classifier in a stepwise manner. The validation set was then used to evaluate the discriminatory performance between chemoresistant and chemosensitive patients. This cross-validation process was repeated 500 times in order to assess its robustness. The classification performance of the most frequently selected protein combinations were assessed within the whole dataset through the generation of receiver operating characteristic (ROC) curves. This predictive analysis identified a 3-protein combination including transketolase (TKT, P29401), laminin subunit gamma-1 (LAMC1, P11047) and tissue alpha-L-fucosidase (FUCO, P04066), as the best protein classifier of chemotherapy response with an AUC of 0.76 (95% CI 0.64–0.87). This protein combination was followed by another 3-protein combination which contained TKT, LAMC1 and c-reactive protein (CRP, P02741) with an AUC of 0.75 (95% CI 0.64–0.86) (Fig. 2B, C Additional file 7: Table S6 and Additional file 8: Fig. S2A, B).

Because response rates of platinum sensitivity are known to fall in a continuum, we next assessed if both protein combinations (TKT + LAMC1 + FUCO and TKT + LAMC1 + CRP) were also good classifiers when considering the most marginal group of chemosensitive patients. Thus, we compared the ability of our protein combinations to discriminate between chemoresistant and partially chemosensitive patients (i.e. those patients who develop recurrence between 6 and 12 months after the last dose of chemotherapy). These partially chemosensitive patients are still taken into consideration in clinical trials assessing chemotherapy response although recent evidence shows they also benefit from re-treatment with platinum-based regimens [31, 32]. We observed that protein combination TKT + LAMC1 + FUCO was able to discriminate between chemoresistant and partially chemosensitive patients with an AUC of 0.76 (95% CI 0.61–0.91). Contrary, TKT + LAMC1 + CRP had worse discriminatory ability with an AUC value of 0.70 (95% CI 0.53–0.87) (Additional file 8: Fig. S2C). Therefore, the 3-protein combination signature TKT + LAMC1 + FUCO showed the best ability to classify patients into chemoresistant and chemosensitive groups, even when considering the closest subgroups of patients.

### The addition of clinical parameters improves the classificatory ability of the protein signature

Next, we assessed if the addition of clinical information could improve the classificatory performance of our protein biomarker signature. We included relevant clinical data routinely used by clinicians during the assessment of patients diagnosed with HGSC prior to treatment. This data included patients’ age, menopausal status, serum CA125 levels at the time of diagnosis and the decision to treat with either primary cytoreductive surgery or neoadjuvant chemotherapy following clinical, radiological and surgical evaluation. The combined analysis of our protein biomarker signature (AUC 0.76; 95% CI 0.64–0.87) with clinical parameters (AUC 0.75; 95% CI 0.63–0.88) showed that it provided a better classificatory power with an AUC value of 0.82 (95% CI 0.72–0.92), *p*-value = 0.09 (Fig. 2D). Interestingly, this analysis shows how the addition of molecular information, and more concretely the protein signature identified in this work, can substantially improve its classificatory performance provided by clinical data alone (Additional file 9: Table S7).

## Discussion

In this work we have established a new strategy that combines molecular and clinical parameters to predict the response to first-line treatment in HGSC patients (HGSC-1LTR) (Fig. 2E). There are currently no protein biomarkers available at the time of HGSC diagnosis able to predict patients individual response to first-line chemotherapy with carboplatin and paclitaxel. In addition, studies evaluating predictive protein markers in ovarian cancer tissues using proteomic approaches are scarce as highlighted in a recent review by our group [13]. The identification of these predictors of chemotherapy response, as the one presented here, allows the prioritization of platinum-based agents if the disease is sensitive, and the use of alternative treatments if resistant in order to improve patient management. This ability to predict a patient's response is particularly relevant in chemoresistant HGSC because alternative non-platinum based chemotherapy regimens could be considered or clinically evaluated in this subgroup of patients [33]. Additionally, it offers several clinical advantages and aid informed clinical decisions. Clinicians could address patients' individual needs and improve their quality of life by avoiding the use of futile treatments. It could also alleviate the economic burden of the healthcare system associated with the use of ineffective treatments. Although understanding why platinum-resistance occurs is essential for improving survival, new strategies able to discriminate between chemoresistant and chemosensitive patients at the time of cancer diagnosis are paramount for better HGSC patient management. Our findings are therefore an important step in advancing towards a stratified risk-management of HGSC patients through the identification of biological predictors of treatment response.

One of the main challenges in this type of projects is the selection of samples to be used for the discovery of protein candidates relevant to our study. In our study we chose tissues for two main reasons. Firstly, archival FFPE tumour samples represent a valuable resource for studying cancer biomarkers because they are widely available in hospitals and are associated to important clinical information (e.g. histology, response to treatment and outcomes). Secondly, all patients included in the study, and for whom the predictive biomarker is intended to be used (i.e. patients with newly diagnosed advanced stage HGSC), undergo a biopsy prior to starting chemotherapy treatment. Therefore, patients’ tissues are available without the need of additional invasive procedures. Moreover, tumour tissues are where potential protein biomarkers are more likely to be enriched. Although FFPE tissues have traditionally been associated to a high variation in protein quality due to formalin-induced chemical modifications and differences in storage times [34], recent studies have confirmed that proteomes are preserved to a comparable extent to those obtained from fresh frozen tissues and are not influenced by their storage [35, 36]. In the study by Coscia et al. [11] authors evaluated 25 advanced stage HGSC FFPE samples by discovery proteomics and identified CT45 as a platinum sensitivity mediator in ovarian cancer. We were able to observe an increased protein abundance by targeted proteomics in chemosensitive samples, in line with the results observed by Coscia et al*.* However, this protein was excluded from subsequent predictive analysis because its targeted peptides were not consistently detected.

Clinical parameters routinely recorded by healthcare professionals can be relevant to improve the classificatory ability of protein biomarkers. An example of this is the Risk of Ovarian Malignancy Algorithm (ROMA) which integrates patients menopausal status to serum CA125 and HE4 levels, to distinguishing between a benign and malignant pelvic mass [37]. We assessed the classificatory ability of our protein signature in combination with relevant clinical data such as the patients age, menopausal status, serum CA125 levels and the decision to treat with either primary cytoreductive surgery or neoadjuvant chemotherapy. Indeed, the addition of the patients data that is currently considered in clinical practice, improved the classification power of the protein biomarker combination. Although mutations in BRCA and other homologous recombination genes are known to predict response to chemotherapy, current clinical guidelines recommend referral for genetic testing at the time of ovarian cancer diagnosis. Hence, since the genetic test is not widely available in all clinical settings until chemotherapy has commenced, a predictor of first-line treatment response, at present, cannot include the genetic information. Although we have not integrated the BRCA mutational status in our HGSC-1LTR strategy, it would be a basic parameter to add in future validation phases together with the use of targeted therapies in the maintenance setting given the rapid advances in the field. In addition, our study was limited by the availability of HGSC samples and the quality of the FFPE tissues for protein extraction and MS analysis. This limitation is associated to differences in patients’ age between the chemoresistant and chemosenstivie groups and TFIp between the discovery and the verification cohorts. Given that we included patients undergoing either primary cytoreductive surgery or interval surgery, it would also be interesting to evaluate the HGSC-1LTR strategy in future studies comparing the molecular characteristics of tissues before and after neoadjuvant chemotherapy. Finally, for the widespread implementation of our signature in routine clinical practice, we would require validation in a higher number of patients and the availability of mass spectrometry technology in the clinics.

## Conclusions

We have established a new strategy (HGSC-1LTR) that combines tissue levels of proteins TKT, LAMC1 and FUCO, together with patients’ age, menopausal status, serum CA125 levels, and treatment approach to predict the response to first-line treatment in HGSC patients. These data are obtained from ready-available biopsies in hospitals, and ready-available clinical data at the time of HGSC diagnosis, thus no additional interventions are required beyond current clinical practice. Because predictive tools are currently lacking for patients with advanced HGSC, this new strategy is clinically relevant for the prediction of chemoresistant patients. Identification of chemoresistance at the time of diagnosis can facilitate the study of alternative treatments aimed at improving the outcome for these patients. In addition, those patients classified as chemosensitive could undergo standard care with platinum-based agents. Therefore, the HGSC-1LTR strategy can allow optimization of therapeutic decision making and individualize HGSC patients’ care.

## Supplementary Information

**Additional file 1: Table S1.** Clinical data of the 21 patients included in the discovery cohort.

**Additional file 2: Table S2.** Clinical data of the 63 patients included in the verification cohort.

**Additional file 3: Table S3.** Discovery cohort quantitative matrix.

**Additional file 4: Figure S1.** Protein quantitative assay development. **(A)** Histogram showing the number of proteins quantified in each group. In red are proteins quantified in at least 4 out of 7 patients which were included in the statistical analysis. **(B)** Peptide identity confirmation between parallel reaction monitoring (PRM) elution profiles of endogenous peptides and internal standards. Transitions showing interferences were removed (middle panel, marked arrow) and those measurements considered to be under the limit of detection were replaced by the background value (lower panel). **(C)** Coefficients of variation (CV) of the 52 peptides showing the low technical variability of the assay.

**Additional file 5: Table S4.** Parallel reaction monitoring quantitative results.

**Additional file 6: Table S5.** Quantitative values (log2 areas) per protein and patient used in the prediction analysis.

**Additional file 7: Table S6.** Performance of the predicted proteins and their ability to classify patients into chemoresistant and chemosensitive groups.

**Additional file 8: Figure S2.** Prediction ability of the protein signatures when considering partially chemosensitive patients and clinical variables. **(A)** Retention time drift of TKT, LAMC1, FUCO and CRP endogenous peptides for all samples analysed. **(B)** Receiver operating curves of the two best protein combination. TKT + LAMC1 + FUCO with an AUC of 0.76 (95% CI 0.64—0.87) and TKT + LAMC1 + CRP with an AUC of 0.75 (95% CI 0.64–0.86). **(C)** Receiver operating curves of the two best protein combination classifiers and their ability to discriminate between chemoresistant and partially chemosensitive patients. TKT + LAMC1 + FUCO with an AUC of 0.76 (95%CI 0.61–0.91) and TKT + LAMC1 + CRP with an AUC value of 0.70 (95% CI 0.53–0.87).

**Additional file 9: Table S7.** Performance of the clinical data included in the exploratory analysis and their ability to classify patients into chemoresistant and chemosensitive groups.

## Data Availability

The datasets generated and analysed during the current study are available in the ProteomeXchange Consortium via the PRIDE [38] partner repository with the dataset identifier PXD035084, https://www.ebi.ac.uk/pride/

## Abbreviations

AUC: Area under the curve
FFPE: Formalin-fixed paraffin-embedded
HGSC: High-grade serous ovarian carcinoma
PARP inhibitor: Poly (ADP-ribose) polymerase inhibitor
PRM: Parallel reaction monitoring
ROC curves: Receiver operating characteristic curves
ROMA: Risk of Ovarian Malignancy Algorithm
TFIp: Treatment-free interval to platinum
