# Supplementary figures and images for: A combination of molecular and clinical parameters provides a new strategy for high-grade serous ovarian cancer patient management
Source: J Transl Med. 2022 Dec 21;20:611. doi: 10.1186/s12967-022-03816-7 (PMC9773449; doi:10.1186/s12967-022-03816-7)

Supplementary Figure 1

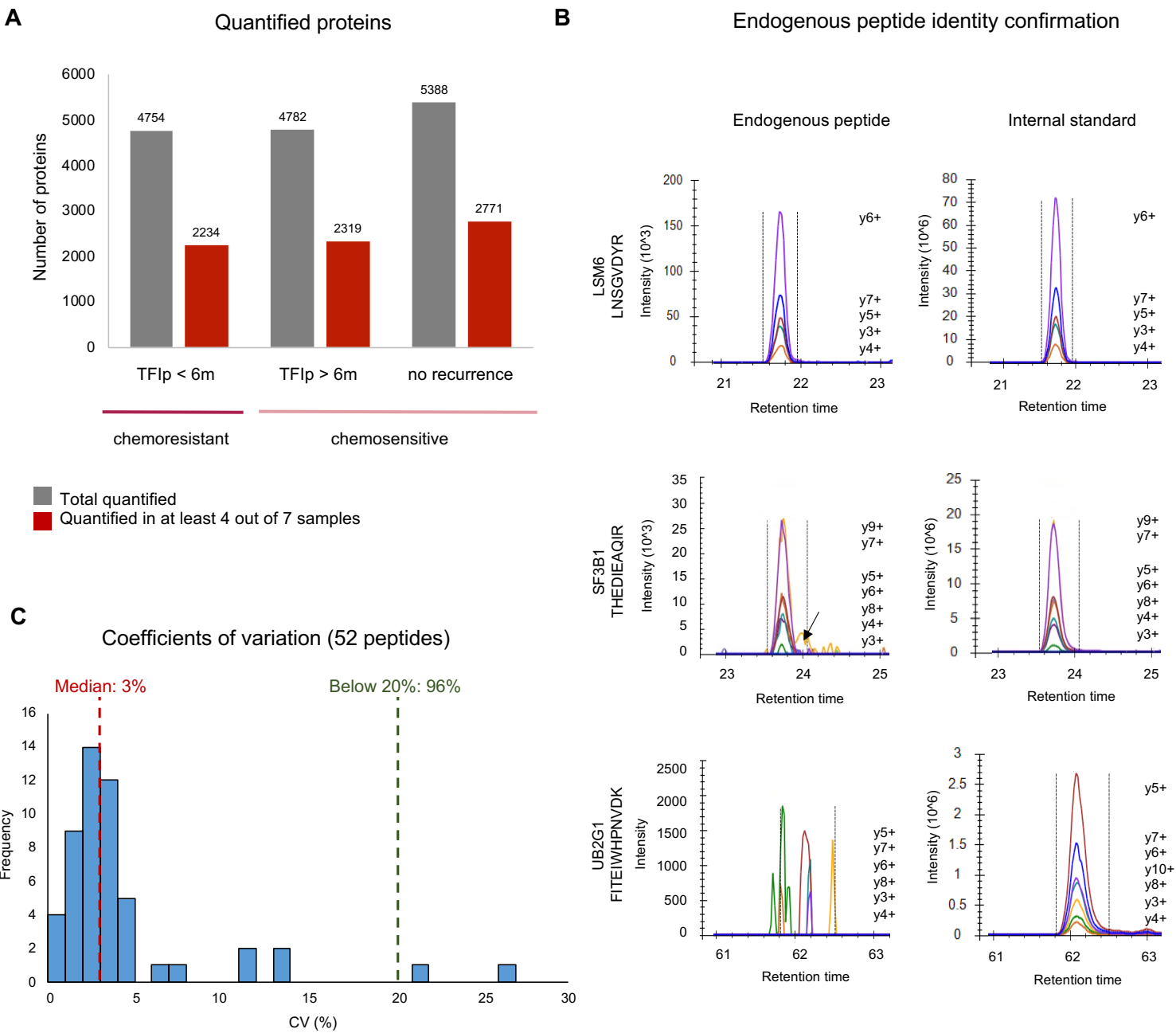

Supplement: Supplementary file 4 — Additional file 4: Figure S1. Protein quantitative assay development. (A) Histogram showing the number of proteins quantified in each group. In red are proteins quantified in at least 4 out of 7 patients which were included in the statistical analysis. (B) Peptide identity confirmation between parallel reaction monitoring (PRM) elution profiles of endogenous peptides and internal standards. Transitions showing interferences were removed (middle panel, marked arrow) and those measurements considered to be under the limit of detection were replaced by the background value (lower panel). (C) Coefficients of variation (CV) of the 52 peptides showing the low technical variability of the assay. [file 12967_2022_3816_MOESM4_ESM.pdf]
