# Supplementary material for: A combination of molecular and clinical parameters provides a new strategy for high-grade serous ovarian cancer patient management
Source: J Transl Med. 2022 Dec 21;20:611. doi: 10.1186/s12967-022-03816-7 (PMC9773449; doi:10.1186/s12967-022-03816-7)

Supplementary Figure 2

A

Peptide retention times

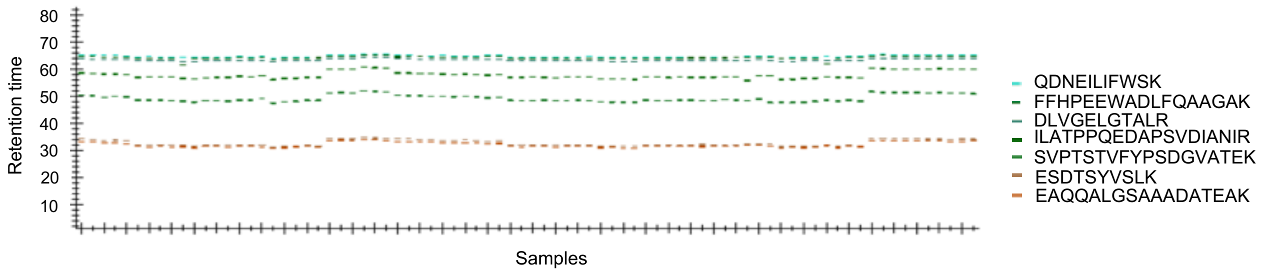

B

Chemoresistant (TFIp < 6m) vs chemosensitive (TFIp > 6m)

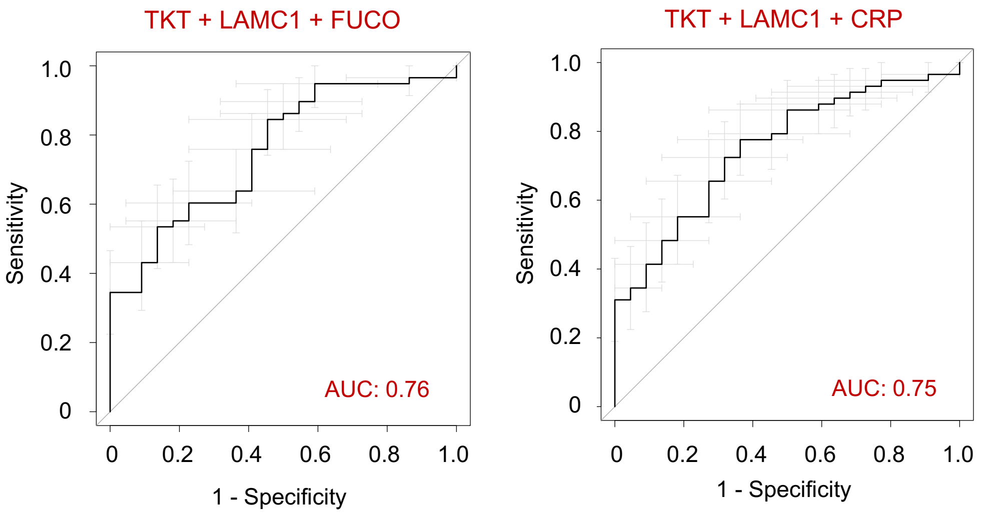

C

Chemoresistant (TFIp < 6m) vs partially chemosensitive (TFIp 6 – 12m)

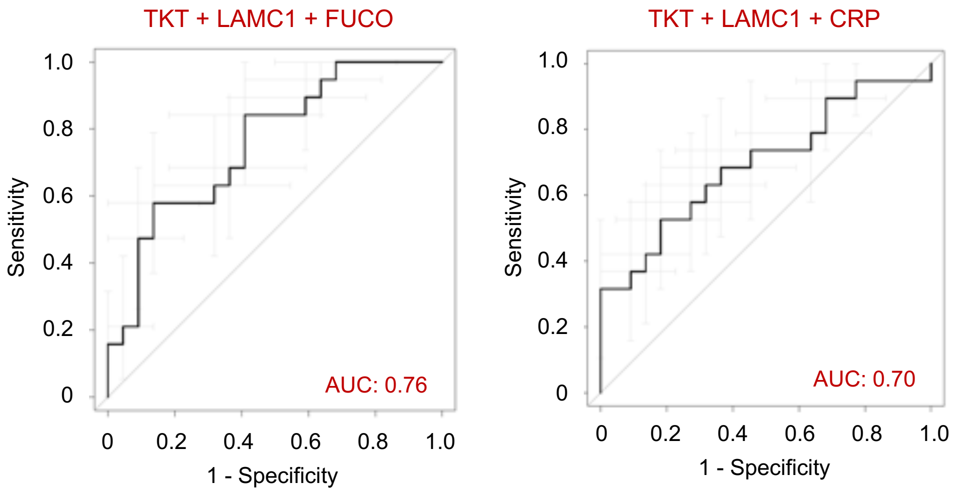

Supplement: Supplementary file 8 — Additional file 8: Figure S2. Prediction ability of the protein signatures when considering partially chemosensitive patients and clinical variables. (A) Retention time drift of TKT, LAMC1, FUCO and CRP endogenous peptides for all samples analysed. (B) Receiver operating curves of the two best protein combination. TKT + LAMC1 + FUCO with an AUC of 0.76 (95% CI 0.64—0.87) and TKT + LAMC1 + CRP with an AUC of 0.75 (95% CI 0.64–0.86). (C) Receiver operating curves of the two best protein combination classifiers and their ability to discriminate between chemoresistant and partially chemosensitive patients. TKT + LAMC1 + FUCO with an AUC of 0.76 (95%CI 0.61–0.91) and TKT + LAMC1 + CRP with an AUC value of 0.70 (95% CI 0.53–0.87). [file 12967_2022_3816_MOESM8_ESM.pdf]
